# SIRPα expression delineates subsets of intratumoral monocyte/macrophages with different functional and prognostic impact in follicular lymphoma

**DOI:** 10.1038/s41408-019-0246-0

**Published:** 2019-10-14

**Authors:** Ya-Ping Chen, Hyo Jin Kim, Hongyan Wu, Tammy Price-Troska, Jose C. Villasboas, Shahrzad Jalali, Andrew L. Feldman, Anne J. Novak, Zhi-Zhang Yang, Stephen M. Ansell

**Affiliations:** 1 Division of Hematology and Oncology, Department of Internal Medicine, National Cheng Kung University Hospital, College of Medicine, National Cheng Kung University, Tainan, Taiwan; 2 Division of Hematology, Department of Medicine, Mayo Clinic, Rochester, MN USA; 3 Department of Immunology, Medical College, China Three Gorges University, Yichang, Hubei China; 4 Department of Laboratory Medicine and Pathology, Mayo Clinic, Rochester, MN USA

**Keywords:** Translational research, Tumour immunology

## Abstract

Signal regulatory protein-α (SIRPα) is a key member of the “do-not-eat-me” signaling pathway, but its biological role and clinical relevance in B-cell NHL is relatively unknown. Using biopsy specimens from follicular lymphoma (FL), we identified three subsets (CD14^+^SIRPα^hi^, CD14^−^SIRPα^low^, and CD14^−^SIRPα^neg^) of monocyte/macrophages (Mo/MΦ) based on CD14 and SIRPα expression. CD14^+^SIRPα^hi^ cells expressed common Mo/MΦ markers; exhibited characteristic differentiation, migration, and phagocytosis; and suppressed T-cell function. CD14^−^SIRPα^low^ cells expressed fewer typical Mo/MΦ markers; migrated less and phagocytosed tumor cells less efficiently; and stimulated rather than suppressed T-cell function. Interestingly, the CD14^−^SIRPα^neg^ subset expressed distinct Mo/MΦ markers compared to the other two subsets; had limited ability to migrate and phagocytose; but stimulated T-cell function. When using SIRPα-Fc to block the interaction between SIRPα and CD47, alone or in combination with rituximab, phagocytosis of tumor cells was differentially increased in the three Mo/MΦ subsets. Clinically, increased numbers of CD14^+^SIRPα^hi^ cells were associated with an inferior survival in FL. In contrast, increased numbers of the CD14^−^SIRPα^low^ subset appeared to correlate with a better survival. Taken together, our results show that SIRPα expression delineates unique subsets of intratumoral Mo/MΦs with differing prognostic importance.

## Introduction

In B-cell non-Hodgkin lymphoma (NHL), particularly follicular lymphoma (FL), two immune signatures have been identified to be associated with patient outcomes^1^. One immune signature that is mediated by T cell is usually associated with a favorable patient outcome^2–5^. The other immune signature that is driven by monocytes/macrophages (Mo/MΦs) generally correlates with an inferior prognosis^6–8^. These tumor-associated macrophages (TAMs) accumulate in the tissue due to monocyte recruitment or due to local proliferation of the tissue-resident macrophages^9,10^. Chemokines and cytokines, including CCL2 (MCP-1), CCL5, and M-CSF are involved in monocyte recruitment^11–13^. While it is thought that TAMs may represent up to 50% of the tumor mass^14^, identification of the markers specific to TAMs has been proved to be challenging as the number of cells expressing well-known markers for Mo/MΦs is significantly low, often negligible, in tumor tissues. The finding that cell surface markers for Mo/MΦs are not static, but instead dynamic, typically reflects changes in their activation status^15^. Although CD68 expression is commonly used to define TAMs, CD68 is an intracellular molecule and is not useful when single-cell isolation of Mo/MΦs is required. In this regard, finding additional ways to define all TAMs is important.

Signal regulatory protein α (SIRPα) is a receptor-like transmembrane protein and signaling through SIRPα on macrophages suppresses both their phagocytic function and inflammatory signaling, thereby contributing to the control of inflammation after infection^16^. The extracellular region of SIRPα comprises three immunoglobulin (Ig)-like domain and cytoplasmic regions containing immunoreceptor tyrosine-based inhibitory motifs, which recruit and activate SH-2 domain-containing phosphotyrosine phosphatases SHP-1 and SHP-2^16^. CD47, another immunoglobulin superfamily protein, is a ligand for SIRPα. In NHL, CD47 is abundantly expressed on the malignant B-cell surface and interacts with SIRPα on the professional phagocytes such as macrophages^17^. The signaling due to interaction of CD47 and SIRPα has been identified as a “self” or so-called “don’t eat me” signal for normal cells to avoid auto-attack by phagocytes. Notably, it has been shown that tumor cells use this mechanism to protect themselves by expressing high levels of CD47^18,19^.

Biological and preclinical relevance of interaction of SIRPα and CD47 has been extensively investigated. However, the studies that characterize SIRPα-expressing Mo/MΦs, especially in the tumor microenvironment, are lacking, possibly due to a low number of Mo/MΦs defined by well-known markers. In the present study, we developed an enrichment protocol to maximize the recovery of Mo/MΦs from the biopsy specimens of B-cell NHL. Using the enriched cells, we defined three subsets of Mo/MΦs that are delineated by SIRPα and CD14 expression. We have characterized these three subsets by phenotype and function (polarization, migration, T-cell suppression, and phagocytosis) and found that they represent distinctly different populations with differing prognostic impact and differing biological responses to antibody treatment.

## Materials and methods

Detailed methods are provided in the online version of this paper.

## Results

### Expression of CD14 on intratumoral Mo/MΦs does not correlate with CD68 expression in FL

**Fig. 1:**
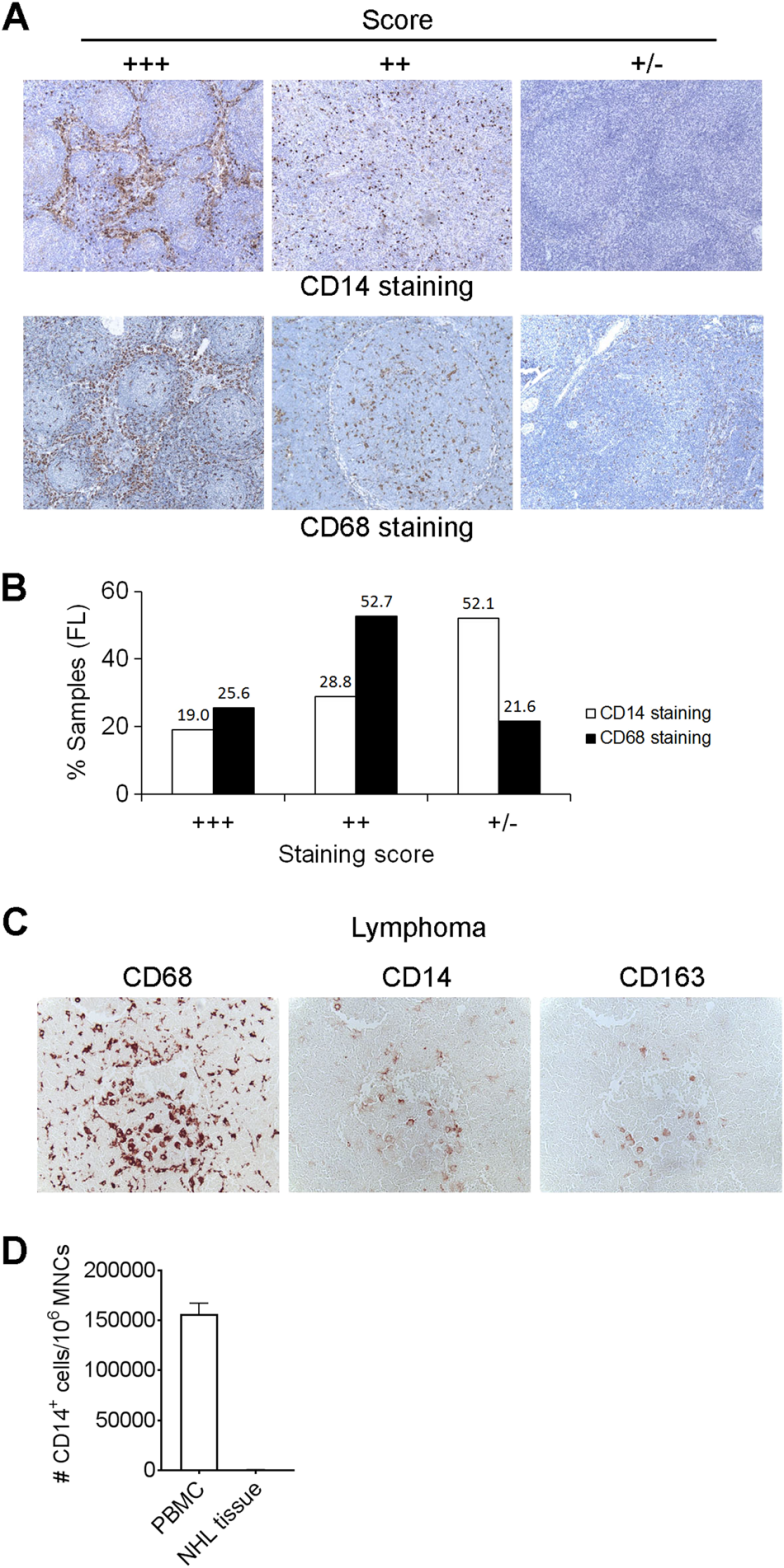
Expression of CD14 on intratumoral Mo/MΦs does not correlate with CD68 expression in FL. **a** Representative images from immunohistochemistry showing staining of CD14 and CD68 with different scores of +++, ++, or +/− in FL patients. **b** Graph showing percentage of patient samples with different scores of CD14 and CD68 staining. *N* = 159. **c** Images of immunohistochemistry from a representative patient biopsy specimen showing staining of CD68, CD14, and CD163. **d** Graph showing cell recovery after CD14-positive selection from PBMCs and NHL tissue. Cell recovery was measured by counting cells before and after CD14-positive selection

To investigate whether CD14^+^ Mo/MΦs are a similar population to Mo/MΦs identified by CD68 expression, Mo/MΦs from biopsy specimens of FL were quantified by immunohistochemistry using CD14 and CD68 expression. Patients with abundant, modest or negligible expression of CD14 or CD68 were given scores of +++, ++, or +/−, respectively (Fig. 1a). In a cohort of 159 FL patients, we observed that most patients (52.1%) were scored as +/− for CD14 expression (Fig. 1b), suggesting a significantly low number or lack of intratumoral CD14^+^ Mo/MΦs in the majority of FL patients. In contrast, CD68 staining was quite visible and a low score of +/− for CD68 expression was found in less than 22% FL patients. CD68 scores with either +++ or ++ accounted for most cases (25.6% and 52.7%, respectively).

Although CD14 expression was abundant in some patient samples, the number of CD14^+^ cells was a rare event in the tumor microenvironment of the vast majority of lymphoma patients. To further determine whether CD14 expression is only present in a subset of Mo/MΦs, we also stained lymphoma sections for CD163, a high-affinity scavenger receptor also expressed on Mo/MΦs. Figure 1c shows a typical staining of CD14, CD68, and CD163 from a representative patient. While CD68 staining was abundant, CD14 staining level was markedly low when compared to CD68 staining. CD163 had a similar staining levels and location to CD14. Supporting this finding, we found that a very small CD14^+^ cell population was present in tissue biopsies compared to the prominent typical CD14^+^ monocyte population found in peripheral blood by flow cytometry. Using CD14 beads to isolate Mo/MΦs from tissue sections resulted in far fewer Mo/MΦs than from the peripheral blood (Fig. 1d).

### SIRPα expression delineate intratumoral Mo/MΦs in FL

**Fig. 2:**
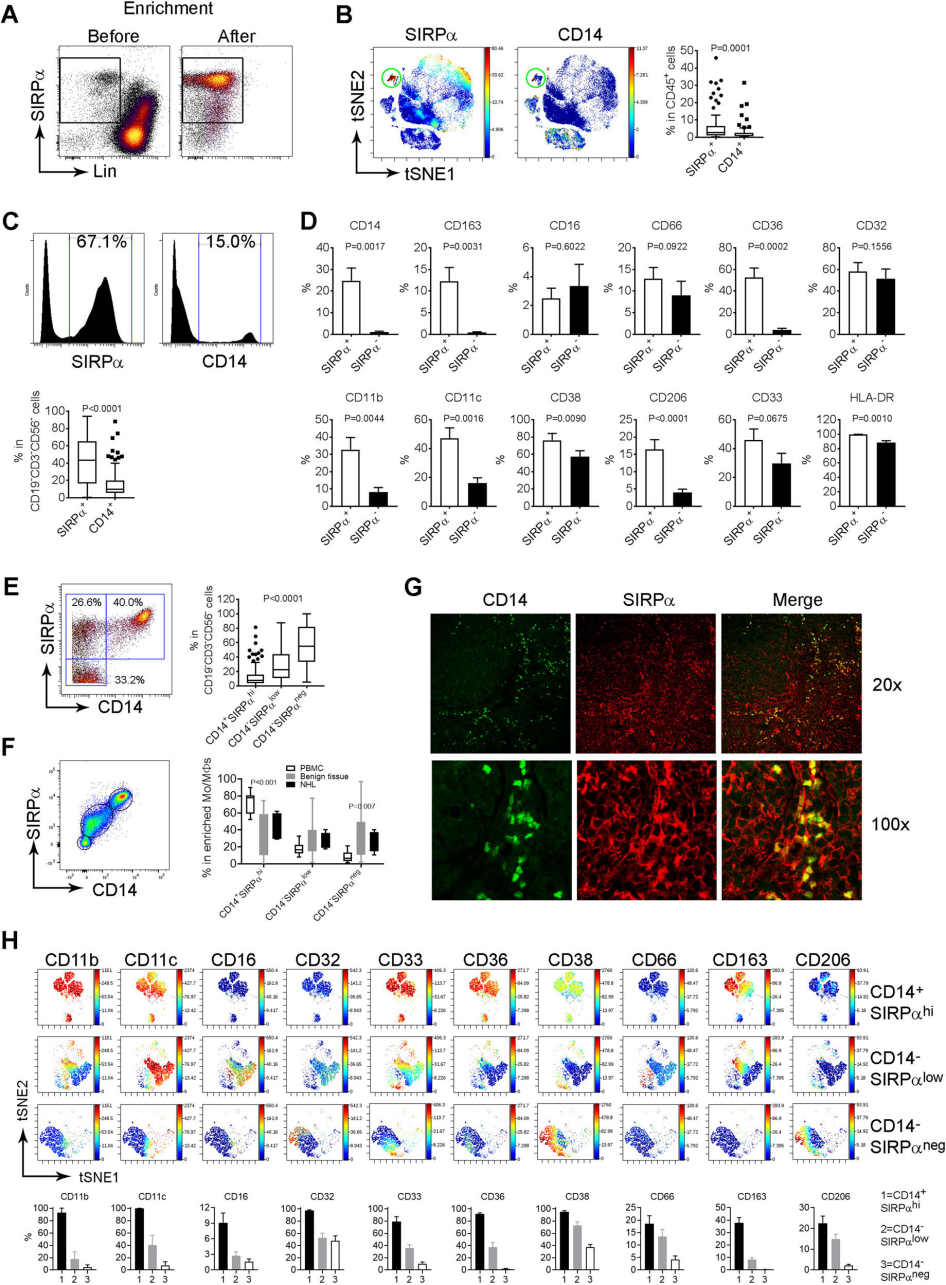
SIRPα expression delineates intratumoral Mo/MΦs. **a** Dot plots showing SIRPα expression on Lin^−^ cells before and after enrichment. **b** The viSNE plots showing expression of SIRPα and CD14 on CD45^+^ cells from a representative patient biopsy specimen of FL. Graph (right) showing percentage of SIRPα^+^ and CD14^+^ cells in CD45^+^ cell population. **c** Histogram plots showing expression of SIRPα and CD14 on CD45^+^CD19^−^CD3^−^CD56^−^ cells from a representative FL biopsy specimen. Graph (right) showing percentage of SIRPα^+^ and CD14^+^ cells in CD45^+^CD19^−^CD3^−^CD56^−^ cells. **d** Graphs showing percent expression of various markers on SIRPα^+^ and SIRPα^+^ cells. **e** Dot plots showing coexpression of SIRPα and CD14 on CD45^+^CD19^−^CD3^−^CD56^−^ cells from a representative patient biopsy specimen of FL. Graph (right) showing percentage of CD14^+^SIRPα^hi^, CD14^-^SIRPα^low^, and CD14^−^SIRPα^neg^ cells within the CD45^+^CD19^−^CD3^−^CD56^−^ cell population. **f** Dot plots showing coexpression of CD14 and SIRPα after Mo/MΦs enrichment. Graph (right) showing percentages of CD14^+^SIRPα^hi^, CD14^−^SIRPα^low^, and CD14^−^SIRPα^neg^ cells in peripheral blood (PBMC), benign lymph nodes and lymphoma tissue (NHL) after enrichment. *P* values indicate the comparison between PBMC and NHL. **g** Representative images of immunofluorescent staining showing localization of CD14^+^ and SIRPα^+^ cells in FL lymphoma tissue. **h** The viSNE plots showing expression of surface markers from CD14^+^SIRPα^hi^, CD14^−^SIRPα^low^, and CD14^−^SIRPα^neg^ cells in FL. Graphs (below) showing the summarization of percentage of the three subsets (CD14^+^SIRPα^hi^, CD14^−^SIRPα^low^, and CD14^−^SIRPα^neg^) expressing each marker (*n* = 13)

Given an extremely low frequency of CD14^+^ cells in biopsy specimens of B-cell NHL, we tested whether SIRPα was able to serve a marker for intratumoral Mo/MΦs. By flow cytometry, we found that SIRPα was highly expressed on Lin^-^ cells but could also be expressed on Lin^+^ cells at a lower level (Fig. 2a). Monocyte enrichment depleted Lin^+^ cells and resulted in a significant enrichment of SIRPα^+^ in Lin^−^ cells. Using CyTOF, we found that SIRPα was abundantly expressed on the Mo/MΦ population that was CD14^+^. However, consistent with the findings from immunohistochemistry, SIRPα was expressed on the Mo/MΦ population to a far greater degree than CD14. The median frequency of SIRPα^+^ and CD14^+^ Mo/MΦs was 2.68% (range: 0.1–45.8%, *n* = 82) and 0.92% (range: 0.1–31.5%, *n* = 82) in CD45^+^ cells, respectively (Fig. 2b). When gating on CD19^−^CD3^−^CD56^−^ cell population, the number of SIRPα^+^ and CD14^+^ Mo/MΦs rose to 43.7% (range: 0.19–94.1%, *n* = 82) and 9.6% (range: 0.01–69.1%, *n* = 82), respectively (Fig. 2c). These results suggest that the number of intratumoral SIRPα^+^ cells is more prevalent than intratumoral CD14^+^ T cells in FL.

We then characterized the phenotype of intratumoral SIRPα^+^ cells compared to SIRPα^−^ cells using Mo/MΦ-related markers. As shown in Fig. 2d, while a negligible amount of SIRPα^−^ cells expressed CD14 and CD163, approximately 20.7% of SIRPα^+^ cells expressed these two classic Mo/MΦ markers. In addition, compared to SIRPα^-^ cells, the number of SIRPα^+^ cells expressing Mo/MΦ markers CD36 (scavenger receptor class B member 3), CD11b (integrin, alpha M, macrophage-1 antigen, and Mac-1), CD11c (integrin and alpha X), CD206 (mannose receptor), and HLA-DR was substantially greater.

Next, we determined whether SIRPα expression identified additional subsets of Mo/MΦs. As shown in Fig. 2e, SIRPα was expressed either independent of or with CD14 on CD19^−^CD3^−^CD56^−^ cells. We observed that the expression level of SIRPα was high when SIRPα was co-expressed with CD14 while expression level of SIRPα was reduced when SIRPα was expressed on non-CD14^+^ cells. This expression pattern of SIRPα and CD14 formed three subsets, named as CD14^+^SIRPα^hi^, CD14^−^SIRPα^low^, and CD14^−^SIRPα^neg^. In vitro enrichment of Mo/MΦs using a negative selection human monocyte enrichment kit plus additional depletion of lymphoma cells produced similar results and the three subsets of Mo/MΦs (CD14^+^SIRPα^hi^, CD14^−^SIRPα^low^, and CD14^−^SIRPα^neg^) were again identified (Fig. 2f). Compared to peripheral blood, lymphoma biopsies had lower numbers of CD14^+^SIRPα^hi^ cells, and increased numbers of CD14^−^SIRPα^neg^ cells, respectively. However, there was no difference between lymphoma biopsies and benign tissue perhaps due to limited sample numbers of benign tissue specimens (*n* = 12 for NHL, *n* = 4 for benign tissue). Immunofluorescent staining in FL lymphoma tissue showed that while the majority of CD14^+^ cells were located around follicles, SIRPα^+^ cells resided both inside and outside of the follicles (Fig. 2g). In agreement with the findings from flow cytometry, SIRPα was co-expressed with CD14 on some cells, presumably the subset of CD14^+^SIRPα^hi^ cells.

By CyTOF, the viSNE analysis on each SIRPα-delineated subset showed that the 3 subsets displayed distinct phenotypes (Fig. 2h). CD14^+^SIRPα^hi^ cells expressed CD11b, CD11c, CD33, CD36, and CD163, whereas CD14^−^SIRPα^low^ subset expressed CD11b, CD11c, CD16, CD33, CD66, and CD163. In contrast, while lacking most surface makers, CD14^−^SIRPα^neg^ cells expressed CD11c, CD32, CD33, CD38, and CD206 at least partially. The percentage of the three subsets expressing each marker is summarized in Fig. 2h (graphs below, *n* = 13). In vitro enriched Mo/MΦs showed that while CD14^+^SIRPα^hi^ cells all expressed CD68, CD163, and CD33, the CD14^−^SIRPα^low^ subset expressed CD68 and CD33 but had low levels of CD163 (Table 1). The CD14^−^SIRPα^neg^ subset also expressed lower levels of CD68 and CD33, and lacked CD163 expression. These results indicate that while CD14^+^SIRPα^hi^ cells had a typical expression profile of Mo/MΦs, CD14^−^SIRPα^low^ and CD14^−^SIRPα^neg^ subsets also displayed some phenotypical features similar to Mo/MΦs.

**Table 1:** Marker expression on subsets of monocytes/macrophages defined by CD14 and SIRPα expression

Marker	CD14^+^	CD14^+^SIRPα^hi^	CD14^−^SIRPα^low^	CD14^−^SIRPα^neg^
CD68	++	++	++	+/−
CD163	++	++	+/−	−
CD33	++	++	+	+/−
CD16	+	+	+	+/−
CD32	++	+	+/−	+
CD64	++	++	+	−
HLA-DR	++	++	++	+
CD206	−	−	−	+/−
CD115	+	n/a	n/a	n/a
CD66c	+/−	n/a	n/a	n/a
CD11b	++	++	+/−	−
CD11c	++	++	++	+/−
CD74	++	n/a	n/a	n/a
S100A8	+	n/a	n/a	n/a
CD63	++	n/a	n/a	n/a
CD106	++	n/a	n/a	n/a
CD37	++	n/a	n/a	n/a
MYH9	++	n/a	n/a	n/a
CD83	−	n/a	n/a	n/a
Siglec-11	++	n/a	n/a	n/a
CD117	++	n/a	n/a	n/a
CD34	−	−	−	+
Lox-1	−	−	−	+

*Note*: ++: high expression; +: moderate expression; +/−: minimal expression; −: no expression; n/a: not assessed

### SIRPα-defined Mo/MΦ subsets display distinct polarization and migration properties

**Fig. 3:**
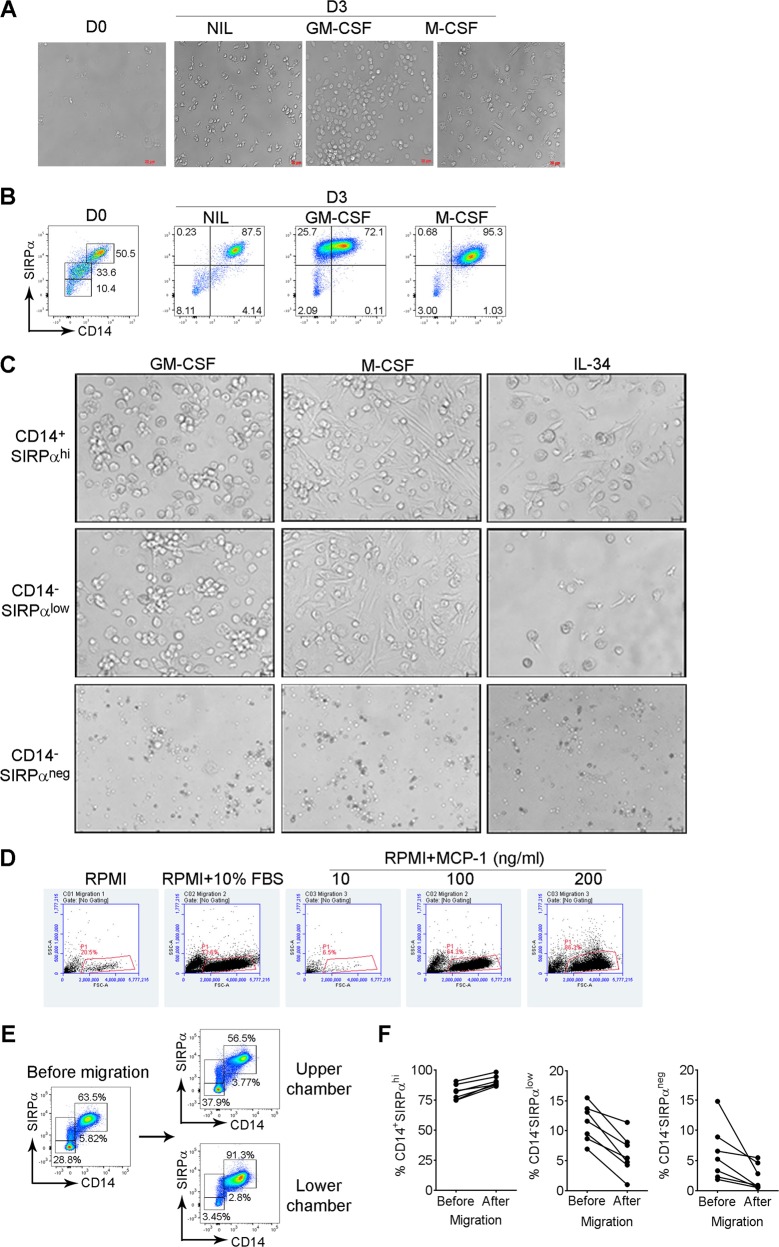
SIRPα-defined Mo/MΦs display distinct polarization and migration properties. **a** Microscopic images showing morphological changes of Mo/MΦs treated with or without GM-CSF or M-CSF for 3 days. **b** Dot plots showing expression of CD14 and SIRPα by Mo/MΦs treated with or without GM-CSF or M-CSF for 3 days. **c** Microscopic images showing morphological changes of CD14^+^SIRPα^hi^, CD14^−^SIRPα^low^ and CD14^−^SIRPα^neg^ cells treated with GM-CSF, M-CSF, or IL-34 for 3 days. **d** Dot plots of SSC and FSC showing the numbers of migrated Mo/MΦs in respond to 10% FBS or escalated doses of MCP-1. **e** Dot plots showing expression of CD14 and SIRPα by Mo/MΦs with (Lower chamber) or without (Upper chamber) migration in response to 10% FBS. **f** Graphs showing percentages of CD14^+^SIRPα^hi^, CD14^−^SIRPα^low^, and CD14^−^SIRPα^neg^ cells in upper (Before) or lower (After) chamber

Enriched Mo/MΦs responded to granulocyte-macrophage colony-stimulating factor (GM-CSF) and M-CSF treatment and underwent clear morphological change as cells became visibly larger (Fig. 3a), suggesting a polarization capacity of these cells. GM-CSF and M-CSF treatment had a differential effect on SIRPα and CD14 expression. GM-CSF treatment reduced the number of CD14^+^ cells, while incubation with M-CSF resulted in a downregulation of SIRPα expression level on enriched Mo/MΦs (Fig. 3b). To assess whether SIRPα-delineated Mo/MΦs subsets possess the ability to polarize when stimulated, we treated the three subsets with cytokines (GM-CSF, M-CSF, and IL-34) for 3 days and monitored cell morphological changes. As shown in Fig. 3c, both CD14^+^SIRPα^hi^ and CD14^−^SIRPα^low^ cells responded to cytokine treatment and underwent visible morphological changes. In contrast, CD14^−^SIRPα^neg^ cells had little response to these three cytokines and the cells did not change morphologically (Fig. 3c).

Using an in vitro migration assay, we added enriched Mo/MΦs to the upper chamber of a transwell and added a chemoattractant (fetal bovine serum (FBS) or monocyte chemoattractant protein-1 (MCP-1/CCL2) to the lower chamber. Cells that migrated into the lower chamber were harvested for cell counting and phenotyping. As shown in Fig. 3d, while there was some migration into the lower chamber with RPMI alone, adding 10% FBS increased Mo/MΦ migration, as cell numbers rose in chamber with 10% FBS compared to chamber with RPMI alone. Similarly, MCP-1, a key chemokine for migration of Mo/MΦs, dose-dependently enhanced the migration of enriched cells when added to the lower chamber. These results suggested that Mo/MΦs from biopsy specimens of B-cell NHL possess the capacity to migrate. Using the same assay, we next measured the migration capacity of each Mo/MΦs subset defined by the expression of CD14 and SIRPα. As shown in Fig. 3e, all three subsets possess migration capacity as all three subsets could be detected in the lower chamber in response to FBS. However, the CD14^+^SIRPα^hi^ subset possessed the highest migration capacity among three subsets as CD14^+^SIRPα^hi^ cells were a dominant population in low chamber (Fig. 3e). When compared to before migration, the relative percentage of the CD14^+^SIRPα^hi^ subset showed the greatest increase in lower chamber (Fig. 3f).

### SIRPα-defined Mo/MΦ subsets possess different abilities to regulate T-cell function, have different cytokine profiles, and exhibit different phagocytosis properties

**Fig. 4:**
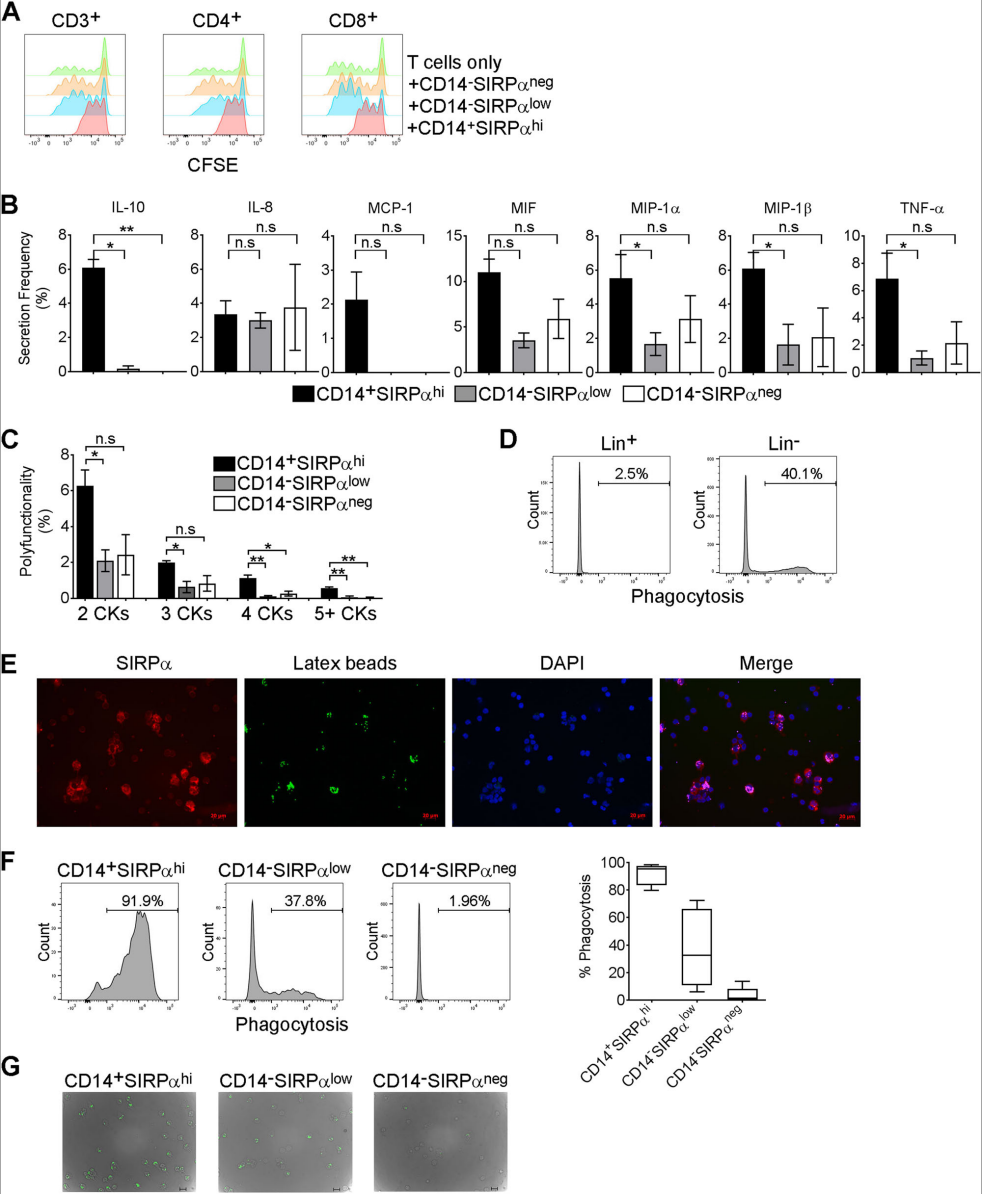
SIRPα-defined Mo/MΦs possess different abilities to regulate of T-cell function and different phagocytosis properties. **a** Overlapped histograms showing CFSE staining of CD3^+^, CD4^+^, or CD8^+^ T cells cocultured with or without CD14^+^SIRPα^hi^, CD14^−^SIRPα^low^ or CD14^−^SIRPα^neg^ cells for 5 days. **b** Graphs showing cytokine/chemokine production by CD14^+^SIRPα^hi^, CD14^−^SIRPα^low^, or CD14^−^SIRPα^neg^ cells determined by the SCBC assay from IsoPlexis system. Results were expressed as the percentage of cells producing cytokines/chemokines. *p < 0.05; **p < 0.01; n.s. no significant difference. *N* = 3. **c** Graph showing multiple cytokine/chemokine production (polyfunctionality) by CD14^+^SIRPα^hi^, CD14^−^SIRPα^low^ or CD14^−^SIRP^αneg^ subset. The numbers of cytokines (2, 3, 4, or 5 plus) produced by single cell from these three subsets of Mo/MΦs were measured by SCBC assay from IsoPlexis system. *p < 0.05; **p < 0.01; n.s. no significant difference. *N* = 3. **d** Histograms showing phagocytosis of Lin^+^ or Lin^−^ cells to latex beads indicated by percentages of cells stained with FITC positive. **e** Fluorescent images showing phagocytosis of latex beads by enriched cells that stained with SIRPα. **f** Histograms showing phagocytosis of CD14^+^SIRPα^hi^, CD14^−^SIRPα^low^, or CD14^−^SIRPα^neg^ cells to latex beads indicated by percentages of cells stained with FITC positive. **g** Fluorescent images showing phagocytosis of latex beads by CD14^+^SIRPα^high^, CD14^−^SIRPα^low^, or CD14^−^SIRPα^neg^ cells

We next determined whether these three subsets differently impacted T-cell proliferation. To do this, freshly flow-sorted CD14^+^SIRPα^hi^, CD14^−^SIRPα^low^, and CD14^−^SIRPα^neg^ cells were incubated with CFSE-labeled CD3^+^, CD4^+^, or CD8^+^ T cells in anti-CD3 antibody-coated plates with anti-CD28 antibody for 5 days. Proliferation of T cells based on CFSE dilution during cell division was analyzed by flow cytometry. As shown in Fig. 4a, after activation and in the absence of Mo/MΦs, a subset of T cells proliferated, with some cells proliferating up to seven cycles. T cells co-cultured with CD14^−^SIRPα^neg^ cells had a similar number of cell divisions, but a larger number of T cells proliferated suggesting that these cells modestly promote T-cell proliferation. Co-culturing T cells with CD14^−^SIRPα^low^ cells modestly reduced T-cell proliferation with a maximum of six proliferations seen. In contrast, cell division was markedly reduced in T cells co-cultured with CD14^+^SIRPα^hi^ cells suggesting that SIRPα^low^ and particularly SIRPα^hi^ cells suppress T-cell function while SIRPα^neg^ cells do not.

Using single‐cell barcode chip (SCBC) assay, we next measured the cytokine/chemokine production profile of these three subsets of Mo/MΦs. As shown in Fig. 4b, 7 of 33 cytokines/chemokines were detectable by at least one subset of Mo/MΦs. There was no difference of cytokine/chemokine production between CD14^−^SIRPα^low^ and CD14^−^SIRPα^neg^ cells. However, we observed that the number of cytokine/chemokine-producing cells was generally higher (significantly higher for some cytokines) in the CD14^+^SIRPα^hi^ subset than in the CD14^−^SIRPα^low^ or CD14^−^SIRPα^neg^ subset (Fig. 4b). Furthermore, the SCBC assay is able to determine the polyfunctionality of three subsets of Mo/MΦs by measuring the numbers of cytokines (2, 3, 4, or 5 plus) produced by single cell. As shown in Fig. 4c, CD14^+^SIRPα^hi^ subset demonstrated the greatest polyfunctionality as the numbers of cells producing 2, 3, 4, or 5 more cytokines were significantly higher in the CD14^+^SIRPα^hi^ subset than in either the CD14^−^SIRPα^low^ or CD14^−^SIRPα^neg^ subset. We did not see a significant difference in polyfunctionality of cytokine production between CD14^−^SIRPα^low^ and CD14^−^SIRPα^neg^ subset.

Mo/MΦs are characterized by their phagocytic capacity that scavenges dead cell debris and infected or transformed cells. We utilized a phagocytosis assay kit to measure phagocytosis by enriched Mo/MΦs. By incubating Mo/MΦs with FITC-labeled latex beads, phagocytosis of cells was measured by flow cytometry or immunofluorescence. As expected, while phagocytosis was negligible in lineage-positive control cells, lineage-negative cells enriched for Mo/MΦs displayed a substantial amount of phagocytosis of latex beads (Fig. 4d). This phagocytosis was confirmed by confocal microscopy in that enriched Mo/MΦs, that stained positive for SIRPα, substantially phagocytosed latex beads (Fig. 4e). To explore phagocytosis by Mo/MΦs subsets delineated by SIRPα, we incubated enriched Mo/MΦs with FITC-labeled latex beads for 3 days and measured phagocytosis of each subset by flow cytometry. As shown in Fig. 4f, CD14^−^SIRPα^neg^ cells displayed very modest phagocytosis as the numbers of cells engulfing latex beads were low. CD14^−^SIRPα^low^ cells displayed moderate phagocytosis capability as there were a substantial number of cells engulfing latex beads. Among the three Mo/MΦ subsets, CD14^+^SIRPα^hi^ cells had the highest phagocytic capacity as almost all the cells were capable of phagocytosing latex beads. Summarized data showed that phagocytosis rates of CD14^+^SIRPα^hi^, CD14^−^SIRPα^low^, and CD14^−^SIRPα^neg^ were 91.3%, 37.1%, and 3.8%, respectively (Fig. 4f). Using flow-sorted cells and a fluorescent assay, we confirmed that CD14^+^SIRPα^hi^, CD14^−^SIRPα^low^ and CD14^−^SIRPα^neg^ cells exhibited differing phagocytic capacity with different numbers of latex beads phagocytosed by each of these three subsets (Fig. 4g).

### Blockade of SIRPα/CD47 signaling enhances phagocytosis of tumor cells

**Fig. 5:**
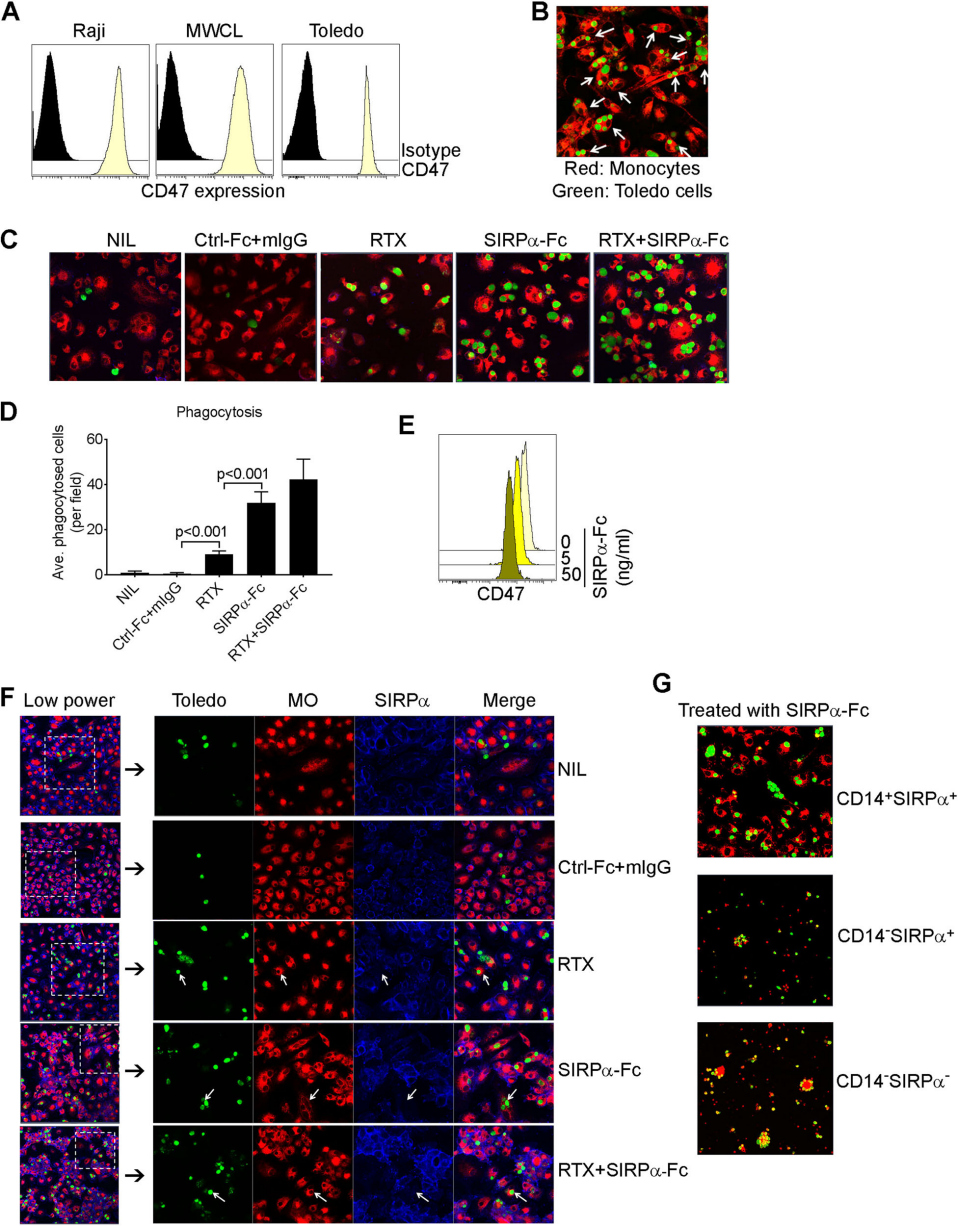
Blockade SIRPα/CD47 signaling enhances phagocytosis of tumor cells. **a** Histograms showing CD47 expression on cell lines Raji (Burkitt lymphoma), MWCL (Waldenstrome macroglubinemia), and Toledo (Diffuse large B-cell lymphoma) cells. **b** Representative fluorescent images showing phagocytosis of Toledo cells (green) by Mo/MΦs (red). Arrows indicate cells engulfing at least one tumor cell. **c** Mo/MΦs were cocultured with Toledo cells in the presence or absence of control (Ctrl) or SIRPα Fc or mIgG and Rituximab (RTX) for 2–4 h and phagocytosis was measured by confocal microscopy. Arrows indicate cells engulfing at least one tumor cells. **d** Graph showing the average number of Mo/MΦs that phagocytosed Toledo cells from five fields of each experiment of multiple samples by confocal microscopy. **e** Histogram showing CD47 expression on Toledo cells treated with different doses of SIRPα-Fc. **f** Representative fluorescent images showing phagocytosis of Toledo cells (green) by Mo/MΦs (red). SIRPα (blue) was stained after phagocytosis assay was completed. Arrows indicated Mo/MΦs engulfing tumor cells without SIRPα (blue) on cell surface. **d** Fluorescent images showing phagocytosis of CD14^+^SIRPα^high^, CD14^−^SIRPα^low^, or CD14^−^SIRPα^neg^ cells treated with SIRPα-Fc

Next, we wanted to test whether the subsets of Mo/MΦs were able to phagocytose tumor cells and whether blocking the interaction between SIRPα and CD47 altered the phagocytosis of each subset. CD47 was highly expressed on lymphoma cells such as Raji (Burkitt lymphoma), MWCL (Waldenstrom macroglobulinemia), and Toledo (Diffuse large B-cell lymphoma) lines when compared to isotype controls (Fig. 5a). We then developed an assay to test the ability of Mo/MΦs to phagocytose lymphoma cells. We found that Mo/MΦs activated with M-CSF and IFN-γ displayed substantial phagocytosis capacity when co-cultured with Toledo cells. Based on multiple microscopic fields analyzed (representative image shown), we found that Mo/MΦs (red) engulfed Toledo cells (green) to variable degrees (Fig. 5b). Some Mo/MΦs engulfed just one Toledo cell and others were able to phagocytose multiple Toledo cells. Using this assay, we investigated whether blocking the interaction between SIRPα and CD47 using SIRPα-Fc enhanced phagocytosis of lymphoma cells by Mo/MΦs. Mo/MΦs were treated with or without SIRPα Fc or control (Ctrl)-Fc for 4 h. As shown in Fig. 5c, untreated or Ctrl-Fc-treated Mo/MΦs displayed a minimal capacity for phagocytosis as the numbers of Mo/MΦs engulfing tumor cells were negligible. However, treatment with SIRPα-Fc markedly enhanced the phagocytosis of Toledo cells by Mo/MΦs as the numbers of Mo/MΦs engulfing Toledo cells increased significantly. A similar effect was seen with Jurkat cells (data not shown). Next, we treated Mo/MΦs with rituximab (RTX), an anti-CD20 chimeric antibody, to compare phagocytosis induced by RTX and SIRPα-Fc. As shown in Fig. 5c, RTX treatment also enhanced the phagocytic capacity of Mo/MΦs. Of note, it appeared that the ratio of lymphoma cells to macrophages was much higher in the RTX, SIRPα-Fc and RTX/SIRPα-Fc groups. The reason for the different ratio in different groups was that unengulfed lymphoma cells were washed out, resulting in an increased ratio of lymphoma cells to macrophages. In contrast, phagocytosed lymphoma cells remained inside of macrophages and were unable to be washed out, which led to a decreased ratio of lymphoma cells to macrophages. The variable ratio actually was a reflection of the capacity for phagocytosis by macrophages. We counted the number of Mo/MΦs that phagocytosed Toledo cells in five fields from each experiment under confocal microscopy and confirmed that both RTX and SIRPα-Fc treatment significantly enhanced phagocytosis of Toledo cells by Mo/MΦs with a greater effect by SIRPα-Fc when compared to RTX. Treatment with the combination of RTX and SIRPα-Fc displayed an additive effect (Fig. 5d). We also confirmed that SIRPα-Fc treatment downregulated CD47 expression on Toledo cells to account for the finding that SIRPα Fc treatment increased phagocytosis of Toledo cells by Mo/MΦs (Fig. 5e).

RTX in combination with CD47 blockade has shown significant promise in treating B-cell lymphoma^20^. We, therefore, tested the effect of RTX and SIRPα-Fc in combination on phagocytosis by each of the SIRPα-defined Mo/MΦ subsets by two methods. First, we stained the Mo/MΦs for SIRPα expression (blue) after phagocytosis assay was completed to determine whether the SIRPα^−^ subset possessed phagocytosis capacity. As shown in Fig. 5f, both SIRPα^+^ and SIRPα^−^ cells were able to phagocytose Toledo cells, regardless of whether the Mo/MΦs were treated with RTX or SIRPα-Fc. However, it is unclear whether the in vitro maturation process decreased SIRPα expression on Mo/MΦs, resulting in the development of SIRPα^−^ cells. In the second method, CD14^+^SIRPα^hi^, CD14^−^SIRPα^low^ and CD14^−^SIRPα^neg^ were freshly isolated using a flow sorter. As shown in Fig. 5g, these three subsets of Mo/MΦs displayed variable levels of phagocytic capacity when treated with SIRPα-Fc. As expected, CD14^+^SIRPα^hi^ cells showed the greatest degree of phagocytosis as more cells from CD14^+^SIRPα^hi^ subset engulfed Toledo cells when compared to the other two subsets of Mo/MΦs. However, both CD14^−^SIRPα^low^ and CD14^−^SIRPα^neg^ cells showed phagocytic activity, albeit to a lesser extent when compared to CD14^+^SIRPα^hi^ cells. Taken together, these results suggest that blocking the interaction between SIRPα and CD47 by SIRPα-Fc enhanced phagocytosis of Mo/MΦs. Interestingly, despite the lack of SIRPα expression, CD14^−^SIRPα^neg^ cells exhibit increased phagocytosis when CD47 is blocked by SIRPα-Fc, and phagocytosis of rituximab treated Toledo cells by this population is enhanced by the addition of SIRPα-Fc.

### SIRPα-defined Mo/MΦ subsets differentially correlate patient outcomes in FL

**Fig. 6:**
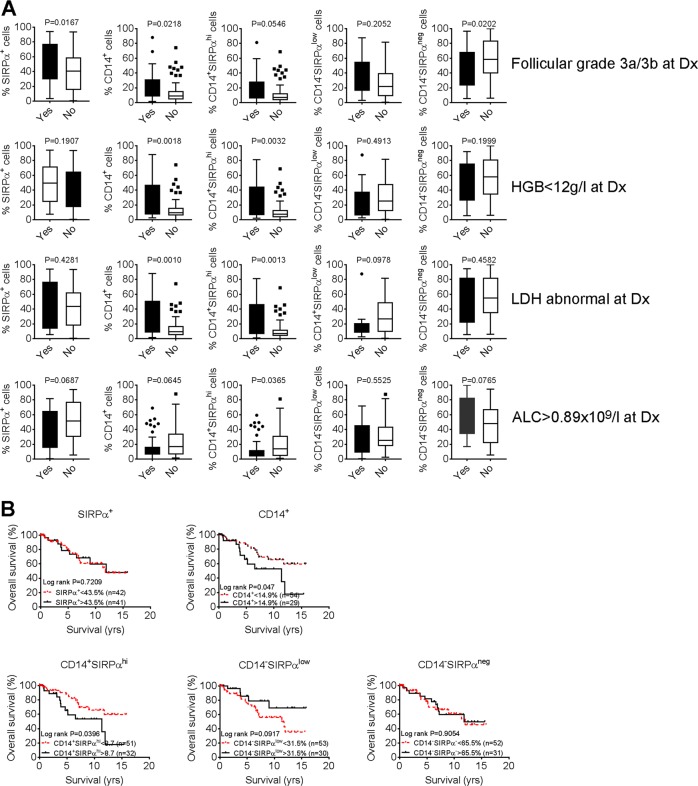
SIRPα-defined Mo/MΦ subsets differentially correlate patient outcomes in FL. **a** Graphs showing percentage of total SIRPα^+^, total CD14^+^, CD14^+^SIRPα^hi^, CD14^−^SIRPα^low^, or CD14^−^SIRPα^neg^ Mo/MΦs in two patient groups. FL patients were grouped using histology (grade 1/2 vs. 3a/3b), hemoglobin (HGB) level (<12 g/dL vs. ≥12 g/dL), lactate dehydrogenase (LDH) level (abnormal vs. not) and absolute lymphocyte counts (ALC, <0.89 × 10^9^/l vs. ≥0.89 × 10^9^/l). **b** Kaplan–Meier curves for overall survival of FL patients (*n* = 83) by the total number of SIRPα^+^, total CD14^+^, CD14^+^SIRPα^hi^, CD14^−^SIRPα^low^, or CD14^−^SIRP^αneg^ Mo/MΦs

Next, we tested whether the presence of SIRPα-defined Mo/MΦ subsets in the tumor microenvironment has an impact on patient outcome in FL. We measured the numbers of total SIRPα^+^, total CD14^+^, CD14^+^SIRPα^hi^, CD14^−^SIRPα^low^, or CD14^−^SIRPα^neg^ Mo/MΦs in biopsy specimens from a cohort of 83 previously untreated FL patients who had been followed for long-term outcome. We observed that intratumoral Mo/MΦ subsets were differentially represented in patient groups defined by clinical parameters. As shown in Fig. 6a, the frequency of total SIRPα^+^ and CD14^+^ cells were significantly higher in patients with follicular grade 1/2 lymphoma by histology at diagnosis (Dx) than patients with FL grade 3a/3b. In patients with hemoglobin (HGB) < 12 g/l at Dx, there were increased numbers of total CD14^+^ or CD14^+^SIRPα^hi^ cells when compared to patients with HGB ≥ 12 g/l at Dx. Similarly, the numbers of total CD14^+^ or CD14^+^SIRPα^hi^ cells increased in patients who had abnormal lactate dehydrogenase (LDH) at Dx when compared to patients whose LDH was normal at Dx. In addition, patients who had an absolute lymphocyte count (ALC) greater than 0.89 × 10^9^/l had reduced CD14^+^SIRPα^hi^ Mo/MΦs when compared to patients who had ALC less than 0.89 × 10^9^/l at Dx. We did not see a difference of frequency of total SIRPα^+^, total CD14^+^, CD14^+^SIRPα^hi^, CD14^−^SIRPα^low^, or CD14^−^SIRPα^neg^ Mo/MΦs correlating with other clinical parameters such as stage (I/II vs. III/IV), B symptoms (yes vs. no), number of nodes (1–3 vs. ≥4), FLIPI scores (1–2 vs. 3–5).

We then determined whether the numbers of intratumoral total SIRPα^+^, total CD14^+^, CD14^+^SIRPα^hi^, CD14^−^SIRPα^low^, or CD14^−^SIRPα^neg^ Mo/MΦ subsets correlated with overall survival in FL patients. As shown in Fig. 6b, while total CD14^+^ cells were associated with a poor overall survival, total SIRPα^+^ displayed no correlation with prognosis in FL. As expected, increased numbers of CD14^+^SIRPα^hi^ cells was significantly associated with a poorer survival in this FL patient cohort. In contrast, we found that the number of CD14^−^SIRPα^low^ cells appeared to be associated with a better outcome in FL patients although a statistically significant difference was not reached (Fig. 6b). Along with the increased numbers of CD14^+^SIRPα^+^ cells (*p* = 0.03), a univariate analysis showed that age >60 years (*p* = 0.039), increased LDH (*p* = 0.015), and the presence of B symptoms (*p* = 0.028) also correlated with overall survival of FL patients. However, a multivariate analysis revealed that age (*p* = 0.029) and B symptoms (*p* = 0.02), but not the number of CD14^+^SIRPα^+^ cells (*p* = 0.16), were independently associated with overall survival in this cohort.

## Discussion

Intratumoral Mo/MΦs play a crucial role in shaping and polarizing the tumor microenvironment, thereby impacting patient outcome in cancers including B-cell NHL^6,7,21–25^. However, in FL, the prognostic impact of intratumoral Mo/MΦs has been variable and is impacted by the specific treatment administered. In the present study, we found SIRPα to be a useful and unique marker to define Mo/MΦ subsets present in biopsy specimens of FL. While it is expressed on all CD14^+^ T cells, SIRPα is also expressed at lower levels on CD14^−^ cells. In addition, CD68^+^ cells without CD14 and SIRPα expression also displayed properties of Mo/MΦs and expressed other Mo/MΦ markers including CD16, CD32, and Lox-1. Previous studies have shown that Mo/MΦs commonly lose CD14 expression particularly when present in tumor tissue and other markers are utilized to define these subsets of Mo/MΦs^6,26,27^. Furthermore, SIRPα expression changes as MΦ become activated^28^, and we show in the present study that CD14 and SIRPα expression on intratumoral Mo/MΦs in FL allows for the definition of three distinct subgroups of cells.

These SIRPα-delineated subsets of Mo/MΦs displayed distinct phenotypical and functional properties. As expected, CD14^+^SIRPα^hi^ cells possessed properties of typical Mo/MΦs including a typical phenotype of Mo/MΦs, being able to migrate in response to MCP-1, phagocytosing latex beads and tumor cells, and suppressing T-cell function. Of note, CD14^+^SIRPα^hi^ cells expressed variable levels of HLA-DR, suggesting that CD14^+^SIRPα^hi^ cells include CD14^+^HLA-DR^lo^ cells, a Mo/MΦs subset typically enriched in lymphoma^21^. Interestingly, CD14^−^SIRPα^low^ cells exhibited properties of typical Mo/MΦs similar to CD14^+^SIRPα^hi^ cells, but lacked CD163 expression, gained CD16 expression, migrated less and phagocytosed tumor cells less efficiently, yet stimulated rather than suppressed T-cell function. The CD14^−^SIRPα^neg^ subset was quite different to the other two subsets and exhibited different Mo/MΦ markers including increased expression of CD32, CD38, and CD206. These cells had limited ability to migrate, and stimulated T-cell function rather than suppressed it. While they had limited ability to phagocytose latex beads, these cells could phagocytose tumor cells. These data confirm that expression of CD14 and SIRPα allow for the identification of cell subsets that exhibit variable phenotypical and functional properties of Mo/MΦs. Existence of three subsets of intratumoral Mo/MΦs identified in the present study is in line with the finding that tissue resident macrophages differentiate in various physiological states^29^, which is supported by the findings that monocytes transform their surface characteristics after infiltration into tissues^30^ and that SIRPα expression was downregulated when co-cultured with tumor cells^31^.

With the advent of cancer immunotherapy, SIRPα/CD47 immunotherapy with an aim to activate the innate immune system has drawn tremendous interest in treating cancer patients^32–34^. Recently, an antibody that blocks the CD47/SIRPα axis (Hu5F9-G4 from Forty seven Inc.) was shown to clinically effective in B-cell lymphomas when given in combination with the anti-CD20 antibody rituximab^20^. Hu5F9-G4 received breakthrough designation from the U.S. Food and Drug Administration for the treatment of lymphoma based on these results^35^. In this study, we find that blockade of CD47 signaling with a decoy receptor SIRPα-Fc promotes increased phagocytosis in all subsets of Mo/MΦs, even the CD14^−^SIRPα^neg^ subset. The CD14^−^SIRPα^neg^ subset of Mo/MΦs is also able to phagocytosis RTX treated cells and the phagocytosis is enhanced when SIRPα-Fc is added. While this may suggest that a low level of SIRPα is indeed present on all Mo/MΦs, it could also be that the Fc portion of both RTX and SIRPα-Fc is recognized by CD32 which is expressed on the CD14^−^SIRPα^neg^ Mo/MΦ subset. Regardless, it is important to note that a significant subset of Mo/MΦs that are typically not appreciated within the tumor microenvironment due to the lack of expression of CD14 and/or SIRPα, are in fact functionally relevant in that they regulate T-cell function and can phagocytose malignant cells particularly in the presence of monoclonal antibodies.

Clinically, we found that an increased number of CD14^+^SIRPα^hi^ cells significantly correlated with a poor prognosis in FL patients, which is in keeping with previous reports that Mo/MΦs are associated with an inferior prognosis in FL patients^1,7^. In contrast, while not statistically significantly different, it appeared that the number of CD14^−^SIRPα^low^ cells is associated with a favorable survival in FL patients. The finding that CD14^-^SIRPα^low^ cells stimulate rather than suppress T cells may explain why CD14^−^SIRPα^low^ cells are associated with a favorable prognosis in FL.

In summary, we identified three subsets of Mo/MΦs based on SIRPα expression that display distinct phenotypes, are functionally different, and have differing prognostic relevance. When exposed to monoclonal antibodies in combination with blockade of CD47/SIRPα-Fc signaling, however, phagocytosis is promoted in all subsets of Mo/MΦs, although to differing degrees. As SIRPα/CD47 blockade is incorporated into clinical practice, the prevalence of subsets of Mo/MΦs defined by SIRPα expression is likely to be important.

## Supplementary information

Supplementary Materials and Methods
