# Supplementary material for: SIRPα expression delineates subsets of intratumoral monocyte/macrophages with different functional and prognostic impact in follicular lymphoma
Source: Blood Cancer J. 2019 Oct 14;9(10):84. doi: 10.1038/s41408-019-0246-0 (PMC6791879; doi:10.1038/s41408-019-0246-0)
Supplement: Supplementary file 1 — Supplementary Materials and Methods [file 41408_2019_246_MOESM1_ESM.docx]

**Supplementary Materials and Methods**

**Patient Samples.** Patients providing written informed consent were eligible for this study if they had a tissue biopsy that on pathologic review showed NHL and had adequate tissue to perform the experiments. Tonsils removed for non-malignant reasons were used as controls. The use of human tissue samples for this study was approved by the Institutional Review Board of the Mayo Clinic/Mayo Foundation.

**Mo/MΦs enrichment and flow cytometry.** Fresh tissues were minced into small fragments by using mechanical dissection of the tissues with a scalpel. Tissue samples were then filtered through a 60μm strainer to create single cell suspensions. Mononuclear cells (MNCs) from tissues or peripheral blood (PB) were isolated by Ficoll-Hypaque density gradient centrifugation. The negative isolation of monocytes/macrophages was performed using the Human Monocyte Enrichment kit with the combination of Human CD19 kits (STEMCELL) to remove malignant B-cells, according to the manufacturer’s instruction, resulting in a purity of Lin^-^ (CD3^-^CD19^-^CD20^-^CD56^-^) cells of more than 95%. Cells were then stained with fluorochrome-conjugated antibodies (Abs) against SIRPα, CD14, CD68, CD45, CD33, CD163, CD64, CD32, CD16, CD3, CD19, CD20, or CD56, or with relevant fluorochrome-conjugated isotype controls, for 30min and analyzed by FACSCanto II flow cytometer (Becton Dickinson, San Jose, CA).

**Mass cytometry (CyTOF).** CyTOF was performed and analyzed as previously described^1,2^. Cells were stained with a cocktail containing 22 metal-tagged monoclonal antibodies designed to interrogate Mo/MΦs subpopulations. Two nucleic acid intercalator probes tagged with iridium (191Ir and 193Ir) were used to identify cellular events. Natural-abundance cisplatin was used as a viability marker. All affinity products tagged to metal isotopes were obtained from Fluidigm (South San Francisco, CA, USA). Specimens were processed in batches to minimize variability and acquired on the CyTOF2^TM^ mass cytometer (Fluidigm). EQ Four Element Calibration Beads (Fluidigm) were used for signal normalization. The CyTOF data were analyzed using online software Cytobank. The viSNE plots of SIRPα-delineated subsets were generated by using viSNE software in the Cytobank.

**Migration assay.** Enriched Mo/MΦs were cultured in Corning Transwell inserts (Corning, NY, USA) in serum-free RPMI-1640 for 4 hours in the presence of chemoattractant (FBS or MCP-1) in the receiver well. Cells that migrated to the lower chamber were collected and quantified using a BD Accuri C6 Plus. Migrated cells were phenotyped by flow cytometry using staining panels that included CD14 and SIRPα antibodies.

**T-cell proliferation assay.** T-cell proliferation was measured by carboxyfluorescein succinimidyl ester (CFSE) staining and calculated by percentage of CFSE^dim^ cells or the number of peaks of CFSE^dim^ cells. Briefly, CD3^+^ T cells were stained with CFSE 500nM for 10 min at RT and quenched with 10% FBS medium. After washing with media three times, T cells were cocultured with CD14^+^SIRPα^hi^, CD14^-^SIRPα^low^ or CD14^-^SIRPα^neg^ Mo/MΦs and CFSE intensity was measured by flow cytometry on day 4.

**Single‐cell barcode chip (SCBC) assay.** Cytokine/chemokine production of Mo/MΦs was measured using SCBC assay from IsoPlexis system (IsoPlexis, Branford, CT). Briefly, Freshly flow-sorted CD14+SIRPαhi, CD14-SIRPαlow and CD14-SIRPαneg Mo/MΦs cells at 1x10^5^/ml were stimulated with 10ng/ml LPS for 24 hours and loaded onto IsoPlexis’s IsoLight micro-chamber that consists of a chip pre-coated with an array of cytokine antibodies (monocyte cytokine panel). The secreted cytokines secreted by activated monocytes were captured onto a corresponding antibody bar and fluoresced into different colors by introducing reagents. The fluorescent bars were scanned and analyzed to measure the numbers of cytokine-producing cells (secretion frequency) and numbers of cytokines produced by single cell (polyfunctionality).

**Phagocytosis assay.** Phagocytosis of fluorescent latex beads by enriched Mo/MΦs was measured using the Phagocytosis Assay Kit according to the manufacturer’s instructions (Cayman Chemical, Ann Arbor, MI). Briefly, latex beads-rabbit IgG-FITC complex was added and incubated with enriched Mo/MΦs at 37°C in humidified air with 5% CO_2_ for 4 hours. Florescent cells (phagocytosed beads) were then determined by flow cytometry on a FACS Canto II.

For cell phagocytosis assay, enriched Mo/MΦs were cultured in X-Vivo-15 media (Lonza) supplemented with 10ng/ml M-CSF (PeproTech) for 7 days and 10ng/ml IFN-γ (PeproTech) for additional 24 hours. Mo/MΦs were then stained with Alexa Fluor 555 (red)-conjugated Wheat Germ Agglutinin (Invitrogen). Tumor cells (Raji (ATCC), MWCL (Mayo Clinic) and Toledo (ATCC)) were labeled with CFSE (green) and cocultured with Mo/MΦs at 1:5 ratio for 2 hours at 37°C in 5% CO_2_ in the presence of 10µg/ml SIRPα-Fc (Trillium Therapeutics, TTI-621) or 10µg/ml negative control-Fc (Trillium Therapeutics, TTI-402). Phagocytosis was assessed by confocal microscopy (Laser scanning microscope 780, Zeiss)^3,4^.

**Statistical analysis.** Statistical analysis was performed using the Student's t test. Significance was determined at p<0.05. To assess the clinical relevance of SIRPα, we determined whether there was a differential prevalence of SIRPα-delineated subsets in patient groups stratified by clinical parameters. We grouped patients using histology (follicular grade 1/2 vs 3a/3b), Ann Arbor stage (I/II vs III/IV), B symptoms (yes vs no), hemoglobin (HGB) level (<12g/dL vs ≥12g/dL), lactate dehydrogenase (LDH) level (abnormal vs not), absolute lymphocyte counts (<0.89x10^9^/l vs ≥0.89x10^9^/l), number of lymph node sites (>4 vs not), FLIPI scores (1-2 vs 3-5) and response (complete or partial) to 1^st^ treatment (yes vs no). To measure overall survival (time from the date of diagnosis to the date of death or last seen), we performed the Kaplan-Meier analysis. The univariate association between SIRPα-delineated subsets and survival was determined with the log-rank test.

1 Yang, Z. Z. *et al.* Expression of LAG-3 defines exhaustion of intratumoral PD-1+ T cells and correlates with poor outcome in follicular lymphoma. *Oncotarget* **8**, 61425-61439, doi:10.18632/oncotarget.18251 (2017).

2 Yang, Z. Z. *et al.* Mass Cytometry Analysis Reveals that Specific Intratumoral CD4(+) T Cell Subsets Correlate with Patient Survival in Follicular Lymphoma. *Cell Rep* **26**, 2178-2193 e2173, doi:10.1016/j.celrep.2019.01.085 (2019).

3 Lin, G. H. Y. *et al.* TTI-621 (SIRPalphaFc), a CD47-blocking cancer immunotherapeutic, triggers phagocytosis of lymphoma cells by multiple polarized macrophage subsets. *PloS one* **12**, e0187262, doi:10.1371/journal.pone.0187262 (2017).

4 Petrova, P. S. *et al.* TTI-621 (SIRPalphaFc): A CD47-Blocking Innate Immune Checkpoint Inhibitor with Broad Antitumor Activity and Minimal Erythrocyte Binding. *Clin Cancer Res* **23**, 1068-1079, doi:10.1158/1078-0432.CCR-16-1700 (2017).
